# Serotype-Specific Acquisition and Loss of Group B *Streptococcus* Recto-Vaginal Colonization in Late Pregnancy

**DOI:** 10.1371/journal.pone.0098778

**Published:** 2014-06-30

**Authors:** Gaurav Kwatra, Peter V. Adrian, Tinevimbo Shiri, Eckhart J. Buchmann, Clare L. Cutland, Shabir A. Madhi

**Affiliations:** 1 Department of Science and Technology/National Research Foundation: Vaccine Preventable Diseases, University of the Witwatersrand, Johannesburg, South Africa; 2 MRC, Respiratory and Meningeal Pathogens Research Unit, University of the Witwatersrand, Johannesburg, South Africa; 3 National Institute for Communicable Diseases: a division of National Health Laboratory Service, Johannesburg, South Africa; 4 Department of Obstetrics and Gynaecology, University of the Witwatersrand, Johannesburg, South Africa; Centers for Disease Control & Prevention, United States of America

## Abstract

**Background:**

Maternal recto-vaginal colonization with Group B *Streptococcus* (GBS) and consequent vertical transmission to the newborn predisposes neonates to early-onset invasive GBS disease. This study aimed to determine the acquisition and loss of serotype-specific recto-vaginal GBS colonization from 20–37+ weeks of gestational age.

**Methods:**

Vaginal and rectal swabs were collected from HIV-uninfected women at 20–25 weeks of gestation age and at 5–6 weekly intervals thereafter. Swabs were cultured for GBS and isolates were serotyped by latex agglutination. Serologically non-typable isolates and pilus islands were characterized by PCR.

**Results:**

The prevalence of recto-vaginal GBS colonization was 33.0%, 32.7%, 28.7% and 28.4% at 20–25 weeks, 26–30 weeks, 31–35 weeks and 37+ weeks of gestational age, respectively. The most common identified serotypes were Ia (39.2%), III (32.8%) and V (12.4%). Of 507 participants who completed all four study visits, the cumulative overall recto-vaginal acquisition rate of new serotypes during the study was 27.9%, including 11.2%, 8.2% and 4.3% for serotypes Ia, III and V, respectively. Comparing the common colonizing serotypes, serotype III was more likely to be associated with persistent colonization throughout the study (29%) than Ia (18%; p = 0.045) or V (6%; p = 0.002). The median duration of recto-vaginal GBS colonization for serotype III was 6.35 weeks, which was longer than other serotypes. Pilus island proteins were detected in all GBS isolates and their subtype distribution was associated with specific serotypes.

**Conclusion:**

South African pregnant women have a high prevalence of GBS recto-vaginal colonization from 20 weeks of gestational age onwards, including high GBS acquisition rates in the last pregnancy-trimesters. There are differences in specific-serotype colonization patterns during pregnancy.

## Introduction

Maternal vaginal colonization with Group B *Streptococcus* (GBS) is the major risk factor for early onset invasive GBS disease (EOD) in newborns [1], [2]. Screening of pregnant women for GBS colonization during the third trimester, coupled with targeted intrapartum antibiotic prophylaxis (IAP) of colonized women during labor, has reduced the incidence of invasive GBS disease in industrialized countries [3].

An alternate preventive strategy against EOD is vaccination of pregnant women, which could enhance transplacental transfer of anti-GBS antibody to the fetus. Studies have identified an association between high maternal serotype-specific anti-capsular polysaccharide (CPS) antibody concentrations with reduced risk of recto-vaginal colonization and reduced risk of newborns developing EOD [4], [5]. Since GBS CPS-protein conjugate vaccines are serotype-specific, it is important to characterize the serotype distribution of GBS in different regions of the world as well as understand the changes which occur in GBS colonization during pregnancy [6]. Other potential vaccine candidates include GBS surface protein antigens such as pilus island (PI) proteins that are present in all GBS isolates [7]. Although it has been shown that maternal GBS colonization during pregnancy may fluctuate [8], [9], [10], there are limited longitudinal studies on the rate of serotype-specific GBS acquisition and duration of colonization during pregnancy.

We aimed to determine the acquisition and loss of GBS recto-vaginal colonization, including serotype-specific changes, among South African pregnant women from 20 weeks to at least 37 weeks of gestational age. We also studied the PI distribution of recto-vaginal colonizing GBS isolates and their association with capsular serotype.

## Materials and Methods

### Study Population

The study was conducted at prenatal community clinics in Soweto (Lillian Ngoyi, Diepkloof, Mofolo and Michael Maponya), Johannesburg from August 2010 to August 2011. Inclusion criteria were HIV-uninfected pregnant women confirmed by HIV ELISA test non-reactivity at enrolment, from 20–25 weeks of gestational age based on last menstrual cycle and who consented to study participation. Exclusion criteria at enrolment included antibiotic treatment in the previous two weeks, any acute illness, symptomatic vaginal discharge and a known or suspected condition in which clinical vaginal examinations were contradicted. If antibiotics were taken after the first visit, the collection of specimens was delayed for at least two weeks after the last antibiotic dose.

### Swab collection and culture of GBS

Lower vaginal and rectal swabs were collected for GBS culture starting at 20–25 weeks (Visit-1), followed by three subsequent visits (Visits 2–4) at 5–6 weekly intervals, up to 37–40 weeks (Visit-4) of gestational age. Demographic and pregnancy-related data were collected at the first visit. All samples were collected by trained study nurses with rayon-tipped swabs that were placed into Amies transport medium without charcoal (cat #MW170, Transwab Amies, Medical wire, U.K.). Swabs were transported to the lab within 4 hours of collection, and processed within 2 hours. For GBS isolation, swabs were inoculated onto CHROMagar StrepB (CA; Media Mage, Johannesburg, South Africa) and the CA plates were incubated at 37°C for 18–24 hours in aerobic conditions [11]. If GBS-like colonies were not visible within 24 hours after incubation, the plates were incubated for a further 24 hours and re-examined for growth. Up to four GBS-like colonies were isolated and confirmed as GBS by testing for CAMP factor, inability to hydrolyze esculin, catalase negativity and group B antigen.

### Capsular serotyping

Serotyping was performed by the latex agglutination method with specific antisera against types Ia, Ib and II to IX CPS antigens (Statens Serum Institute, SSI, Sweden) as described [12]. Isolates that tested negative by latex agglutination for all serotypes were further typed by a PCR method for serotypes Ia, Ib, II, III, IV and V using primer sequences described by Poyart *et al* [13]. The gene encoding dlts was used as a PCR positive control for GBS identification.

### Pilus typing

Pilus island proteins of all GBS isolates were detected by PCR for PI-1, PI-2a and PI-2b, with primers that target the genomic regions coding for the ancillary protein (AP)-1 of each PI. Isolates that tested negative for all the AP1 genes, or isolates from which neither PI-2a or PI-2b could be detected, were amplified by a second set of primers representing conserved regions of AP-2 as described previously [7].

### Statistical analysis

Data were analyzed using SAS version 9.2 software (SAS Institute, Inc., NC, USA). A visit sample pair of vaginal and rectal swabs was considered negative if no GBS growth was evident on either swab, and positive if GBS was grown from either swab. The pregnant women were grouped into transient, intermittent and persistent carriers according to the presence of GBS colonization and to individual serotypes at the four sampling time points. Transient carriers were defined as women who were colonized at only one of the four visits, intermittent carriers as those who were colonized at two or three of the visits and persistent carriers as those colonized at all four study visits.

Descriptive statistics included the prevalence of colonization at individual time points and changes of recto-vaginal colonization status. Analysis of the changes in recto-vaginal colonization over time was restricted to the 507 participants who completed all four study visits. New acquisition of GBS was defined as positive culture of a new serotype which was not previously present. The new acquisition rate was defined as the number of new serotype acquisitions divided by the number of participants who were at a risk of acquiring the new serotype. Thus, women who were already previously colonized by a particular serotype were excluded subsequently from the denominator for estimating acquisition rate for the homotypic serotype. The rate of new acquisitions by all GBS serotypes were calculated from the sum of acquisition rates for the individual serotypes, and by using the above methods for GBS acquisition rates in a serotype independent manner. Clearance of colonization was defined as a negative GBS culture for a specific serotype following a positive sample at the previous visit for the homotypic serotype. The rate of colonization clearance was defined as the number of GBS-negative participants at the analyzed time point divided by the number of participants at the previous visit who were positive for that serotype, and was also calculated in a serotype independent manner.

Survival analysis methods were used to estimate the duration of colonization of specific serotypes. A colonizing event was defined as the period of time between acquisition and clearance of a GBS serotype. Date of acquisition was calculated as the midpoint between the last visit without serotype-specific colonization and the first visit at which a positive sample was obtained for the homotypic serotype, while date for termination of serotype-specific colonization was calculated as the midpoint between the last visit with colonization and the subsequent negative visit for that serotype. In this analysis, if colonization occurred at the first visit, this was taken as the start of colonization, and if colonization occurred at the last visit, a right censoring approach was applied. We used the Kaplan-Meier method to estimate the duration of GBS colonization. The log-rank test was used to examine differences in duration of carriage between serotypes.

Positive predictive value (PPV) and negative predictive value (NPV) were calculated for the culture results at different sampling points with the 37–40 week visit as the reference standard. For participants who were colonized with same serotype on multiple visits, only one serotype specific isolate was used to study PI association with capsular serotype.

The chi-square test was used to compare proportions. Logistic regression analysis was used to determine the association between GBS colonization and demographic characteristics at enrolment. A p-value of <0.05 was considered significant.

### Ethics statement

The study was approved by the Human Research Ethics Committee of the University of the Witwatersrand (IRB/Protocol-M090937) and informed written consent was obtained from all participating mothers. The trial is registered with South African National Clinical Trials Register, number DOH-27-0210-3012.

## Results

### Demographic characteristics

**Figure 1:**
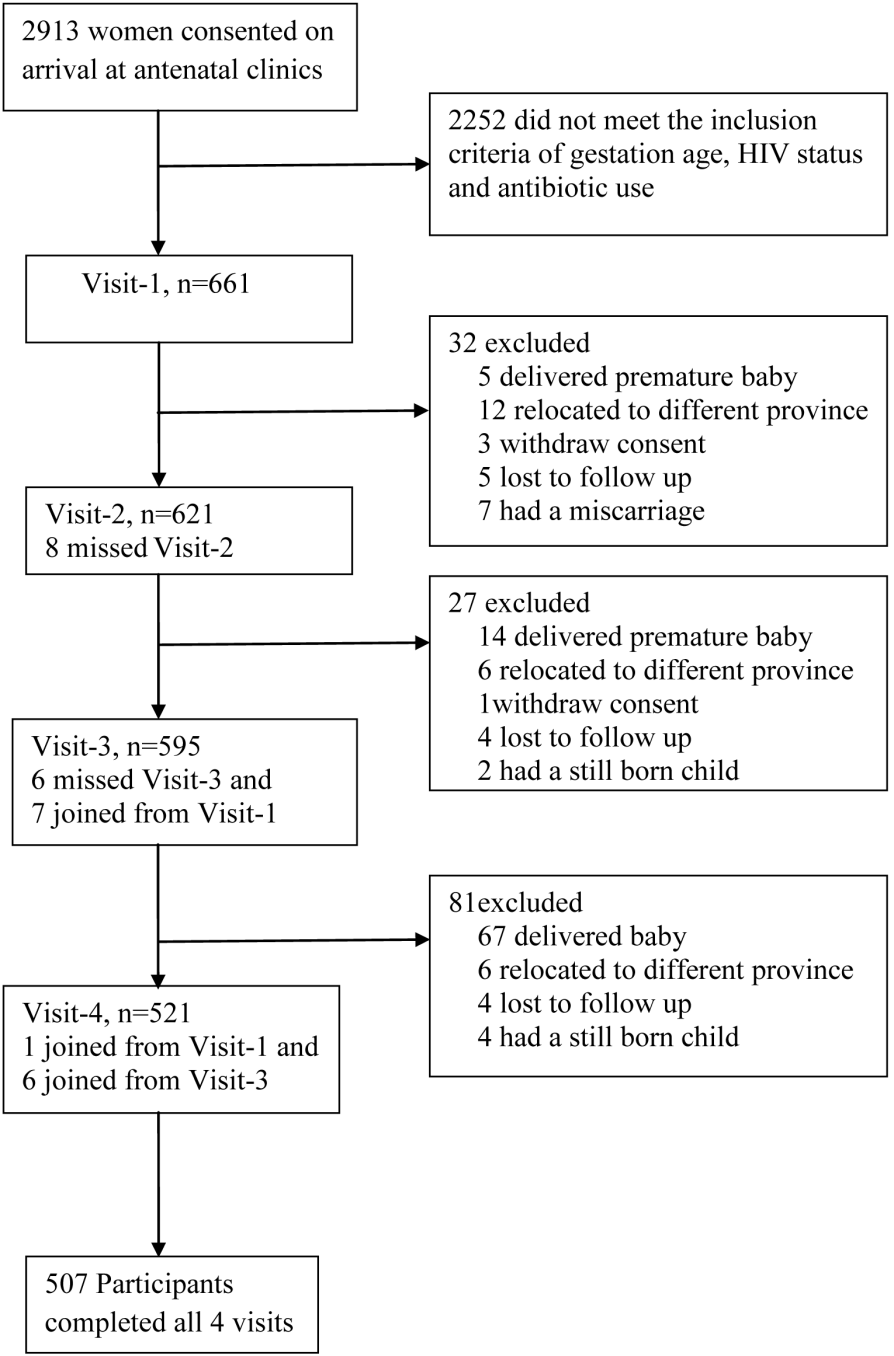
Trial Profile.

Of the 661 enrolled participants, 621 (93.9%), 595 (90.0%) and 521 (78.8%) completed visits 2, 3 and 4, respectively. Five-hundred and seven (76.7%) women completed all four study visits. A detailed trial profile is indicated in figure 1. The main reason for women not attending all four visits was birth of the baby (13%; 86/661) before the final visit. The demographic characteristics are displayed in table 1. The mean age of the participants at enrolment was 25.9 (standard deviation; S.D±5.6) years. Only 5 (0.76%) pregnant women have taken antibiotic treatment during the study.

**Table 1:** Demographics of the study population at time of enrolment (n = 661).

Demographic characteristic		Overall (n = 661)	GBS Colonized	GBS Uncolonized
Age (years)	<20	92 (13.9%)^a^	27 (29.3%)^b^	65 (70.7%)^c^
Mean age: 25.9 (S.D±5.6)	20–24	234 (35.4%)	78 (33.3%)	156 (66.6%)
	25–28	161 (24.4%)	53 (32.9%)	108 (67.1%)
	29–32	95 (14.4%)	29 (30.5%)	66 (69.5%)
	33–35	40 (6.1%)	20 (50.0%)	20 (50.0%)
	36+	39 (5.9%)	11 (28.2%)	28 (71.8%)
Parity	0	338 (51.1%)	97 (28.7%)	241 (71.3%)
Median parity: 0 (range; 0–5)	1–2	304 (46.0%)	113 (37.2%)	191 (62.8%)
	3–5	19 (2.9%)	8 (42.1%)	11 (57.9%)
Gravidity	1	286 (43.3%)	80 (28.0%)	206 (72.0%)
Median gravidity: 2 (range; 1–8)	2	221 (33.4%)	80 (36.2%)	141 (63.8%)
	3	106 (16.0%)	38 (35.8%)	68 (64.2%)
	≥4	48 (7.3%)	20 (41.7%)	28 (58.3%)
Previous Abortion (spontaneous)	0	553 (83.7%)	176 (31.8%)	377 (68.2%)
Median abortion: 0 (range; 0–3)	1	88 (13.3%)	36 (40.9%)	52 (59.1%)
	2	19 (2.9%)	6 (31.6%)	13 (68.4%)
	3	1 (0.2%)	0 (0.0%)	1 (100%)
Stillborn	0	651 (98.5%)	214 (32.9%)	437 (67.1%)
Median stillbirths: 0 (range; 0–1)	1	10 (1.5%)	4 (40.0%)	6 (60.0%)

a Data are no (%) of total participants, ^b,c^Data are row %.

### Prevalence of GBS colonization

The overall prevalence of recto-vaginal GBS colonization was 33.0% (218), 32.7% (203), 28.7% (171) and 28.4% (148) at 20–25 weeks, 26–30 weeks, 31–35 weeks and 37+ weeks of gestational age, respectively. The lower prevalence of colonization associated with 31–35 weeks and 37+ weeks compared to 20–25 weeks and 26–30 weeks was specifically associated with a decrease in prevalence of vaginal colonization, table 2 (23.3% to 19.0%). In the 86 women who gave birth before the final visit, vaginal GBS colonization was detected in 17(19.8%) at the last attended visit compared to 99/521 (19.0%) who gave birth after visit-4 (p = 0.867). The inclusion of rectal swab GBS-culture increased the overall detection of GBS colonization by approximately 10% across the four study time-points (p<0.0001) and the prevalence of rectal colonization remained similar at each study time-point.

**Table 2:** Prevalence of Group B *Streptococcus* colonization during the study visits.

Site of colonization	Visit-1	Visit-2	Visit-3	Visit-4
	(20–25 weeks)	(26–30 weeks)	(31–35 weeks)	37+weeks)
	n = 661	n = 621	n = 595	n = 521
	Mean gestation age: 22.7 weeks	Mean gestation age: 27.9 weeks	Mean gestation age: 32.5 weeks	Mean gestation age: 37.5 weeks
Vaginal only (%; 95% CI)	62	65	47	31
	(9.4%; 7.2–11.6)	(10.5%; 8.1–12.9)	(7.9%; 5.7–10.1)	(5.9%; 3.9–7.9)
Rectal only (%; 95% CI)	64	62	56	49
	(9.7%; 7.5–12.0)	(10%; 7.6–12.4)	(9.4%; 7.1–12.0)	(9.4%; 6.9–11.9)
Both vaginal andrectal (%; 95% CI)	92	76	68	68
	(13.9%; 11.3–16.5)	(12.2%; 9.63–14.8)	(11.4%; 8.9–14.0)	(13.1%; 10.2–16.0)
Vaginal and/or rectal(%; 95% CI)	218	203	171	148
	(33.0%; 29.4–36.6)	(32.7%; 29.0–36.4)	(28.7%; 25.1–32.3)	(28.4%; 24.5–32.3)

CI-Confidence interval, n = number of participants.

Of several demographic characteristics evaluated at enrolment independently by univariate analysis, parity (OR: 1.22; 95% CI: 1.02–1.47; p = 0.030) and gravidity (OR: 1.17; 95% CI: 1.01–1.36; p = 0.046), (Table 3) were significantly associated with GBS recto-vaginal colonization, with the highest colonization prevalence observed among women with parity ≥3 (42.1%) and gravidity of ≥4 (41.7%), (Table 1). In a multivariate analysis, none of the demographic characteristics were found to be associated with GBS recto-vaginal colonization, (Table 3). In a serotype-specific univariate analysis at enrolment, multiparity was associated with a higher prevalence of serotype III colonization (OR: 1.37; 95% CI: 1.07–1.76; p = 0.012), gravidity also showed possible association with serotype III colonization (p = 0.068). In the multivariate analysis parity was found to be associated with serotype III colonization (Adjusted OR: 6.69; 95% CI: 1.47–30.4; P = 0.014). Gravidity (p = 0.053) and abortions (p = 0.057) also showed a possible association with serotype III colonization, (Table 3). None of the demographic characteristics were found to be associated with serotype Ia colonization in the univariate or multivariate analysis. There were no identifiable factors associated with a higher prevalence of colonization with GBS at visit-4 alone.

**Table 3:** Univariate and multivariate association between serotype-specific colonization and observed demographic characteristics at enrolment.

Characteristic	Overall GBS colonization at enrolment		Serotype III colonization at enrolment		Serotype Ia colonization at enrolment	
	Univariate	Multivariate	Univariate	Multivariate	Univariate	Multivariate
	OR (95% CI), p	AOR (95% CI), p	OR (95% CI), p	AOR (95% CI), p	OR (95% CI), p	AOR (95% CI), p
Age	0.98 (0.95–1.02),	1.01 (0.97–1.06),	0.98 (0.92–1.03),	1.00 (0.96–1.04),	0.99 (0.94–1.04),	
	0.536	0.527	0.45	0.96	0.656	
Parity	1.99 (0.83–4.79),	1.37 (1.07–1.76),	6.69 (1.47–30.4),	1.05 (0.82–1.34),	0.71 (0.27–1.90),	
	0.121	0.012	0.014	0.69	0.499	
Gravidity	0.63 (0.27–1.50),	1.22 (0.99–1.51),	0.22 (0.05–1.02),	1.11 (0.91–1.35),	1.48 (0.57–3.81),	
	0.296	0.068	0.053	0.317	0.422	
Abortion	1.77 (0.73–4.30),	1.10 (0.66–1.81),	4.14 (0.96–18.0),	1.25 (0.82–1.91),	0.86 (0.31–2.38),	
	0.207	0.721	0.057	0.298	0.774	
Stillborn	1.25 (0.32–4.94),	0.94 (0.12–7.51),	0.76 (0.08–7.30),	2.60 (0.66–10.3),	2.14 (0.49–9.29),	
	0.741	0.951	0.813	0.171	0.309	

OR: odds ratio; AOR: adjusted OR; CI: confidence interval.

### Serotype and pilus island distribution

The proportional representation of serotypes remained consistent at each of the consecutive sampling time-points. Of women colonized, the proportional representation of the major serotypes were 36.2% to 41.4% for Ia, 31.3% to 34.9% for III, 10.3% to 15.6% for V, 7.2% to 7.5% for II, 3.5% to 4.6% for Ib, 2.0% to 4.0% for IV and 0.0% to 3.3% for IX (Table S1 in file S1). The concordance of serotypes for GBS cultured concurrently from vaginal and rectal swabs was 91.3%, 89.5%, 94.1% and 94.1% for the four consecutive visits, respectively. Only 1.6% of GBS isolates were serologically non-typable by latex agglutination and were serotyped by PCR.

All GBS isolates harbored one or more PIs, either PI-2a on its own or with a combination of PI-2a or PI-2b in combination with PI-1.The most common PI arrangement was PI-2a on its own, which occurred in 103/227 (45.4%), 92/211 (43.6%), 79/175 (45.1%) and 63/152 (41.5%) of isolates at visits 1–4, respectively, followed by PI combination PI-2b and PI-1, which occurred in 75/227 (33.0%), 69/211 (32.7%), 68/175 (38.9%) and 58/152 (38.2%) of isolates at visits 1–4, respectively. The least common PI arrangement was a combination of PI-2a and PI-1 which occurred in 49/227 (21.6%), 50/211 (23.7%), 29/175 (16.0%) and 31/152 (20.4%) of isolates at visits 1–4, respectively. There were no significant changes in the prevalence of PI distribution with respect to different visits, with the exception of PI-2a which was less common at visit-4 (18.0%, 94/521) compared to visit-1 (23.0%, 152/661; p = 0.007), and which was attributable to a lower prevalence of serotype Ia at visit-4 (10.6%, 55/521) compared to visit-1 (14.2%, 94/661).

**Figure 2:**
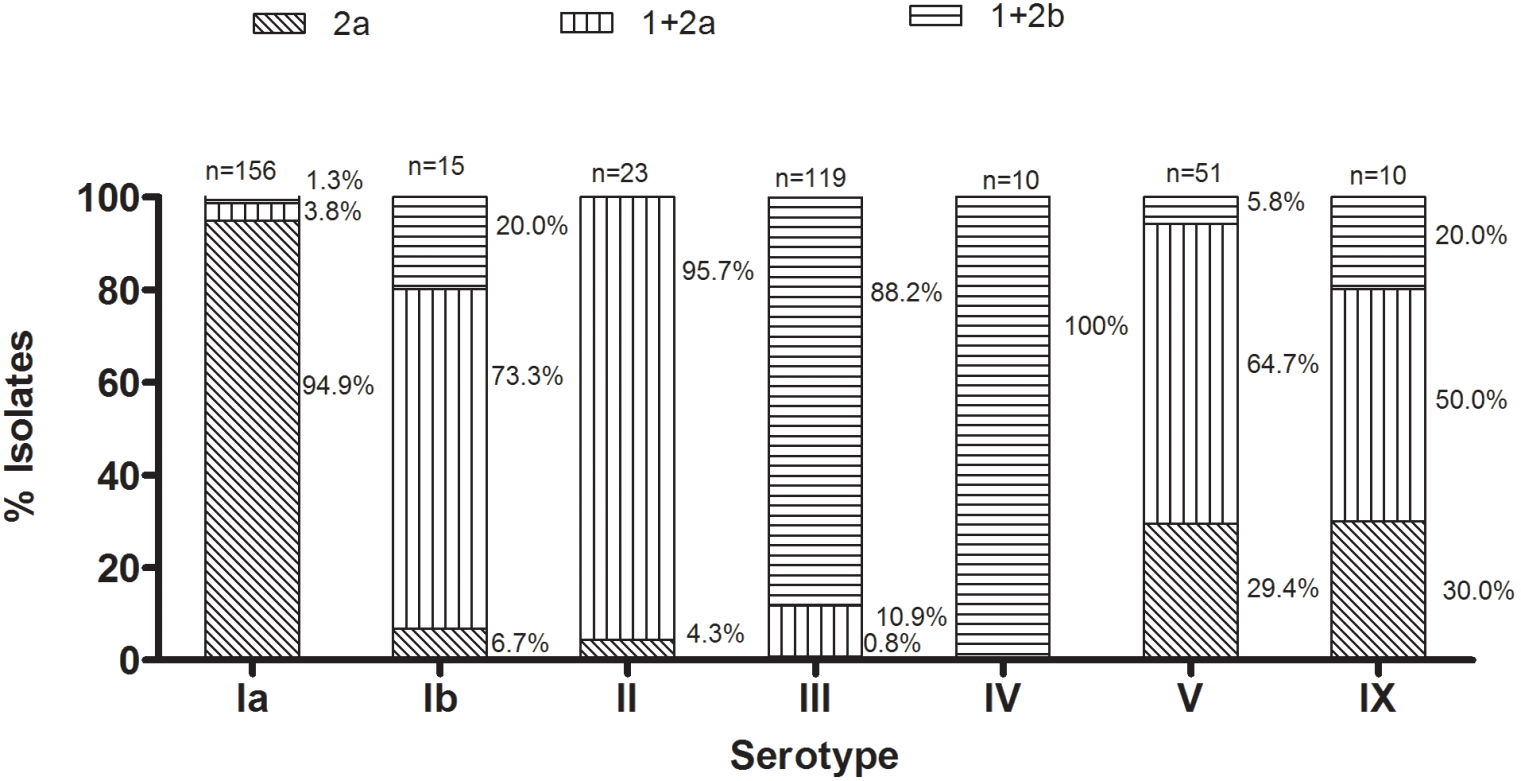
Association of pilus island proteins and serotypes among Group B *Streptococcus* isolates.

There was a strong correlation between the presence of particular combinations of PI and the serotype; Figure 2. Most serotype Ia isolates were associated with PI-2a (94.9%; 148/156), whereas the majority of serotype III isolates were associated with the combination of PI-1 and PI-2b (88.2%; 105/119). The association between PIs and serotype V was more variable, with a PI-1+PI-2a combination occurring in 64.7% (33/51) and PI-2a alone occurring in 29.4% (15/51) of isolates.

### Changes in GBS colonization overtime

Five hundred and seven participants who completed all four study visits were similar in their demographic characteristics compared to the 154 participants not included in this analysis (data not shown). In the analyzed subset, the prevalence of recto-vaginal GBS colonization was 32.1% (163), 30.4% (154), 29.0% (147) and 27.8% (141) at 20–25 weeks, 26–30 weeks, 31–35 weeks and 37+ weeks of gestational age, respectively. Two hundred and fifty-two (49.7%) women were colonized at least once during the study period, of whom 70 (27.8%) were persistent carriers, 83 (32.9%) transient carriers and 99 (39.3%) were intermittently colonized for any serotype (Table S2 in file S1).

The cumulative serotype-specific prevalence across the study period was 23.7% (120/507) for Ia, 18.3% (93/507) for III, 7.1% (36/507) for V, 4.3% (22/507) for II and 2.8% (14/507) for Ib. All GBS serotypes were variable in their colonization patterns. Comparing the three most common colonizing serotype carriers, 29% (27/93) of serotype III carriers were associated with persistent colonization compared to serotype Ia (18%; 21/120; p = 0.045) or V (6%; 2/36; p = 0.002). Serotype V was the most dynamic, with 94.4% (34/36) of colonized women either being transient or intermittent carriers compared to 82.5% (99/120; p = 0.106) for Ia and 71.0% (66/93; p = 0.004) for III. Only one serotype was detected in 83.3% (210/252) of GBS carriers during the study period, with 85.7% (60/70) of women persistently colonized being associated with the same serotype.

Of the 16.7% (42/252) women in whom multiple serotypes were detected over the study period, two serotypes were detected in 9.5% (24/252) and three serotypes in 7.1% (18/252) participants. Among women in whom multiple serotypes were detected, a new serotype was observed at the immediate next visit in 85.7% (36/42) of cases, while a new serotype was detected following a period of no colonization by the preceding serotype in six women.

### New acquisition and clearance of colonization

Three hundred and forty-four participants who completed all four study visits were not colonized at visit-1, of whom 89 (25.9%) became colonized at one of the subsequent three visits. When including new serotype acquisition in those previously colonized by a heterotypic serotype (n = 39), the cumulative overall recto-vaginal acquisition rate of new serotypes during the study, calculated from the sum of acquisition rates for the individual serotypes was 27.9%. The number of new acquisitions was highest for serotypes Ia (11.2%, 49/436), III (8.2%, 37/451) and V (4.3%, 21/492); table S2 in file S1. The mean new acquisition rate of GBS was 11.4% (S.D ±0.5%) at 5–6 week visit intervals, including 11.6% between visit-1 and visit-2, and 10.8% and 11.7% in the intervals of subsequent consecutive visits. Of 163 participants who were colonized at visit-1, 76 (46.6%) were no longer colonized by visit-4. The rate of colonization-clearance was 75% (6/8) for serotype Ib, 73.3% (11/15) for V and 63.4% (45/71) for Ia. The overall clearance of any GBS colonization was 30.1% (49/163), 29.2% (45/154) and 32.7% (48/147) between visits-1 and -2, visits-2 and -3, and visits-3 and -4, respectively. No demographic characteristics were identified that were associated with either new acquisition or clearance of colonization.

### Duration of GBS colonization

The median duration of recto-vaginal GBS colonization was 6.35 weeks for serotype III, which tended to be longer than other serotypes, including serotype Ia (median: 5.21 weeks; p = 0.02; table 4) which was the second most common colonizing serotype. The difference in duration of colonization between serotype III and less prevalent serotypes was not statistically significant.

**Table 4:** Estimated duration of Group B *Streptococcus* recto-vaginal colonization.

Serotype	Colonization duration (weeks)*		
	Mean (95% CI)	Median	p-value†
Ia	7.52 (6.6–8.4)	5.21	0.026
Ib	5.22 (4.03–6.42)	3.62	0.358
II	6.11 (5.07–7.16)	4.24	0.736
III	9.15 (8.1–10.2)	6.35	Reference
IV	6.94 (3.8–10.0)	4.81	0.998
V	8.60 (6.80–10.39)	5.96	0.332
IX	6.21 (4.7–7.7)	4.31	0.651

*Time from enrolment, CI-Confidence interval,

† compared to serotype III.

### Predictive values for each visit culture with respect culture status at visit-4

Positive and negative predictive values of serotype-specific culture at 20–25 weeks, 26–30 weeks and 31–35 weeks of gestational age compared to 37+ weeks colonization status are presented in table 5. The overall positive predictive values were 53.4%, 61.7% and 67.4% for GBS-positive cultures at 20–25 weeks, 26–30 weeks and 31–35 weeks, respectively, relative to positivity at 37+ weeks, while the negative predictive values for 20–25 weeks, 26–30 weeks and 31–35 weeks ranged from 84.3% to 88.3%. Serotypes Ia and V had lower PPVs compared with serotype III at each time-point. The observed PPVs at 31–35 weeks, were 55–70% for the three commonest serotypes.

**Table 5:** Predictive value for 20–25, 26–30 and 31–35 weeks cultures in relation to culture status at 37+ weeks.

Serotype	20–25 weeks		26–30 weeks		31–35 weeks	
	PPV % (95% CI)	NPV % (95% CI)	PPV % (95% CI)	NPV % (95% CI)	PPV % (95% CI)	NPV % (95% CI)
Overall GBS	53.4 (45.4–61.2)	84.3 (80.0–88.0)	61.7 (53.5–69.4)	87.0 (83.0–90.3)	67.4 (59.1–74.9)	88.3 (84.6–91.5)
Ia	36.6 (25.5–48.9)	94.5 (91.9–96.4)	49.2 (36.1–62.3)	95.5 (93.2–97.2)	55.0 (41.7–67.9)	96.2 (94.0–97.8)
Ib	25.0 (3.9–65.0)	99.0 (97.7–99.7)	66.7 (22.7–94.7)	99.4 (98.3–99.9)	75.0 (20.3–95.9)	99.2 (98.0–99.8)
II	38.5 (14.0–68.4)	98.8 (97.4–99.6)	54.6 (23.5–83.1)	99.0 (97.7–99.7)	75.0 (42.8–94.2)	99.6 (98.5–99.9)
III	58.9 (45.0–71.9)	95.8 (93.5–97.4)	62.8 (48.1–75.9)	95.6 (93.3–97.3)	67.9 (53.7–80.1)	96.5 (94.3–98.0)
IV	50.0 (12.4–87.6)	100 (99.2–100)	60.0 (15.4–93.5)	100 (99.2–100)	75.0 (20.3–95.9)	100 (99.3–100)
V	26.7 (8.0–55.1)	97.2 (95.3–98.4)	47.6 (25.8–70.2)	98.4 (96.8–99.3)	57.1 (28.9–82.2)	98.0 (96.3–99.0)
IX	100 (19.3–100)	99.6 (98.6–99.9)	50.0 (12.4–87.6)	99.8 (98.9–100)	100 (30.5–100)	99.8 (98.9–100)

PPV: Positive predictive value, NPV: Negative predictive value, CI-Confidence interval.

## Discussion

To our knowledge this is the first serotype-specific longitudinal study conducted of recto-vaginal GBS colonization in pregnant women, in whom we demonstrated a high prevalence and acquisition rate of GBS recto-vaginal colonization. The overall rate of new acquisition at 5–6 week interval is in agreement with a previous study of non-pregnant women, although, the serotype-specific rates differed [14]. All GBS serotypes were variable in their colonization patterns, possibly due to the complex interaction between immunity and specific GBS serotypes, which is still incompletely understood. It may be that the higher frequency of persistent colonization and longer overall duration of colonization by serotype III, is related to a weaker natural immune response associated with its colonization compared to other serotypes [15]. Consequently, there is a higher risk of exposure at birth to serotype III in our population, which corroborates with it being responsible for 49.2% to 57.7% of EOD in our setting [16], [17]. The higher acquisition rate of serotype Ia may result in there being inadequate time for natural immunity to this serotype developing in the pregnant woman, which consequently increases the newborn’s risk of developing EOD from serotype Ia, associated with 22.6% to 31% of EOD cases in our setting [16], [17].

The high incidence of new acquisition and loss of colonization during pregnancy highlights why screening is required as late as 35–37 weeks’ gestational age for the IAP strategy to be effective, which is concordant with another study in pregnant women [18]. In our study, if women had been screened at 31–35 weeks, 29.1% (42/141) of those who were colonized at 37+ would not have had IAP offered to them and a lesser proportion (13.3%; 48/366) may have unnecessarily received IAP as they were no longer colonized at 37+ weeks. Although we did not identify any demographic characteristics associated with new acquisition or clearance of GBS, additional risk factors such as sexual activity during pregnancy were not fully explored [19]. The PPV of GBS cultures obtained from 20–35 weeks varied in serotype distribution compared to that at 37+ weeks. The prevalence of different GBS serotypes in a particular population can affect the PPV of late antenatal GBS cultures.

The high cumulative prevalence of GBS colonization (49.7%) found in our study is comparable to longitudinal studies from Denmark and Zimbabwe [8], [10]. Furthermore, the prevalence of colonization observed by us at 37+ weeks of gestational age was 28.4% (148/521), which was similar to that reported in cross-sectional studies from Europe [20] and USA [21]. The prevalence of GBS colonization from African countries ranges from 16.5% in Malawi, 21–23% in The Gambia, Ethiopia and Tanzania and 31.6% in Zimbabwe [22], [23], [24], [25], [26]. Our results also showed a decrease in the prevalence of GBS colonization with respect to increase in gestational age. This finding agrees with studies from the USA and Australia [9], but contrasts with others that reported an increase in colonization with increasing gestational age [28], [29].

Our findings on the dominant serotypes are comparable with serotype distribution data of maternal colonizing isolates from industrialized countries, including 13% to 35% for serotype Ia and 15% to 44% for serotype III [6]. The identification of serotype IX in our study was notable in that it is rarely reported in colonizing studies and not previously described in Africa. To our knowledge, only 8 GBS colonizing isolates have been identified as serotype IX, including three from Denmark, two from Germany and one each from Canada, Hong Kong and Australia [30]. Our data on PI distribution is comparable to earlier published studies [7], [31] showing that all GBS isolates carried at least one PI, and were associated with the presence of either PI-2a or PI-2b identified alone or in combination with PI-1.

Our study is limited by the sensitivity of detection of GBS on selective media which is estimated at 85% [11] and by the fact that in most cases only the dominant serotype was determined. This can lead to an underestimation of persistent colonization, an overestimation of new acquisitions, and an underestimation of the duration of carriage.

Recent developments in the clinical evaluation of a tri-valent GBS polysaccharide-protein conjugate vaccine has renewed interest in the potential of this vaccine to protect neonates against invasive GBS disease by reducing recto-vaginal colonization during pregnancy [32]. The findings of this study will be important in considering study design when evaluating the efficacy of maternal GBS vaccination protecting against GBS recto-vaginal acquisition and colonization during pregnancy as surrogate information on clinical vaccine efficacy may be gained by determining the immune responses that correlate with protection against serotype-specific GBS acquisition and colonization during pregnancy.

## Supporting Information

File S1 (DOCX)

## References

[1] Verani JR, McGee L, Schrag SJ (2010) Prevention of perinatal group B streptococcal disease–revised guidelines from CDC, 2010. MMWR Recommendations and reports : Morbidity and mortality weekly report Recommendations and reports/Centers for Disease Control 59: 1–36. 21088663

[2] Chan SHS WK, Lee WH (2000) Review on Group B Streptococcal Infection. HK J Paediatr (New Series) 5: 8.

[3] Schrag SJ, Schuchat A (2004) Easing the burden: characterizing the disease burden of neonatal group B streptococcal disease to motivate prevention. Clinical infectious diseases: an official publication of the Infectious Diseases Society of America 38: 1209–1211. 15127329 10.1086/382889

[4] Baker CJ, Kasper DL (1976) Correlation of maternal antibody deficiency with susceptibility to neonatal group B streptococcal infection. The New England journal of medicine 294: 753–756. 768760 10.1056/NEJM197604012941404

[5] Baker CJ, Edwards MS, Kasper DL (1981) Role of antibody to native type III polysaccharide of group B Streptococcus in infant infection. Pediatrics 68: 544–549. 7033911

[6] Ippolito DL, James WA, Tinnemore D, Huang RR, Dehart MJ, et al (2010) Group B streptococcus serotype prevalence in reproductive-age women at a tertiary care military medical center relative to global serotype distribution. BMC infectious diseases 10: 336. 21106080 10.1186/1471-2334-10-336 PMC3004907

[7] Madzivhandila M, Adrian PV, Cutland CL, Kuwanda L, Madhi SA (2013) Distribution of pilus islands of group B streptococcus associated with maternal colonization and invasive disease in South Africa. Journal of medical microbiology 62: 249–253. 23065545 10.1099/jmm.0.052951-0

[8] Hansen SM, Uldbjerg N, Kilian M, Sorensen UB (2004) Dynamics of Streptococcus agalactiae colonization in women during and after pregnancy and in their infants. Journal of clinical microbiology 42: 83–89. 14715736 10.1128/JCM.42.1.83-89.2004 PMC321715

[9] Goodman JR, Berg RL, Gribble RK, Meier PR, Fee SC, et al (1997) Longitudinal study of group B streptococcus carriage in pregnancy. Infect Dis Obstet Gynecol 5: 237–243. 18476144 10.1155/S1064744997000409 PMC2364546

[10] Mavenyengwa RT, Afset JE, Schei B, Berg S, Caspersen T, et al (2010) Group B Streptococcus colonization during pregnancy and maternal-fetal transmission in Zimbabwe. Acta obstetricia et gynecologica Scandinavica 89: 250–255. 19916889 10.3109/00016340903398029

[11] Kwatra G, Madhi SA, Cutland CL, Buchmann EJ, Adrian PV (2013) Evaluation of Trans-Vag broth, colistin-nalidixic agar, and CHROMagar StrepB for detection of group B Streptococcus in vaginal and rectal swabs from pregnant women in South Africa. J Clin Microbiol 51: 2515–2519. 23698527 10.1128/JCM.00251-13 PMC3719654

[12] Afshar B, Broughton K, Creti R, Decheva A, Hufnagel M, et al (2011) International external quality assurance for laboratory identification and typing of Streptococcus agalactiae (Group B streptococci). Journal of clinical microbiology 49: 1475–1482. 21325542 10.1128/JCM.02365-10 PMC3122801

[13] Poyart C, Tazi A, Reglier-Poupet H, Billoet A, Tavares N, et al (2007) Multiplex PCR assay for rapid and accurate capsular typing of group B streptococci. Journal of clinical microbiology 45: 1985–1988. 17376884 10.1128/JCM.00159-07 PMC1933079

[14] Foxman B, Gillespie B, Manning SD, Howard LJ, Tallman P, et al (2006) Incidence and duration of group B Streptococcus by serotype among male and female college students living in a single dormitory. American journal of epidemiology 163: 544–551. 16421237 10.1093/aje/kwj075

[15] Davies HD, Adair C, McGeer A, Ma D, Robertson S, et al (2001) Antibodies to capsular polysaccharides of group B Streptococcus in pregnant Canadian women: relationship to colonization status and infection in the neonate. The Journal of infectious diseases 184: 285–291. 11443553 10.1086/322029

[16] Madzivhandila M, Adrian PV, Cutland CL, Kuwanda L, Schrag SJ, et al (2011) Serotype distribution and invasive potential of group B streptococcus isolates causing disease in infants and colonizing maternal-newborn dyads. PloS one 6: e17861. 21445302 10.1371/journal.pone.0017861 PMC3061872

[17] Madhi SA, Radebe K, Crewe-Brown H, Frasch CE, Arakere G, et al (2003) High burden of invasive Streptococcus agalactiae disease in South African infants. Annals of tropical paediatrics 23: 15–23. 12648320 10.1179/000349803125002814

[18] Manning SD, Lewis MA, Springman AC, Lehotzky E, Whittam TS, et al (2008) Genotypic diversity and serotype distribution of group B streptococcus isolated from women before and after delivery. Clinical infectious diseases : an official publication of the Infectious Diseases Society of America 46: 1829–1837. 18462173 10.1086/588296 PMC9491394

[19] Meyn LA, Moore DM, Hillier SL, Krohn MA (2002) Association of sexual activity with colonization and vaginal acquisition of group B Streptococcus in nonpregnant women. American journal of epidemiology 155: 949–957. 11994235 10.1093/aje/155.10.949

[20] Motlova J, Strakova L, Urbaskova P, Sak P, Sever T (2004) Vaginal & rectal carriage of Streptococcus agalactiae in the Czech Republic: incidence, serotypes distribution & susceptibility to antibiotics. The Indian journal of medical research 119 Suppl: 84–87. 15232169

[21] Campbell JR, Hillier SL, Krohn MA, Ferrieri P, Zaleznik DF, et al (2000) Group B streptococcal colonization and serotype-specific immunity in pregnant women at delivery. Obstet Gynecol 96: 498–503. 11004347 10.1016/s0029-7844(00)00977-7

[22] Suara RO, Adegbola RA, Baker CJ, Secka O, Mulholland EK, et al (1994) Carriage of group B Streptococci in pregnant Gambian mothers and their infants. The Journal of infectious diseases 170: 1316–1319. 7963736 10.1093/infdis/170.5.1316

[23] Joachim A, Matee MI, Massawe FA, Lyamuya EF (2009) Maternal and neonatal colonisation of group B streptococcus at Muhimbili National Hospital in Dar es Salaam, Tanzania: prevalence, risk factors and antimicrobial resistance. BMC Public Health 9: 437. 19948075 10.1186/1471-2458-9-437 PMC2791767

[24] Musa Mohammed DA, Woldeamanuel Y, Demissie A (2012) Prevalence of group B Streptococcus colonization among pregnant women attending antenatal clinic of Hawassa Health Center, Hawassa, Ethiopia. Ethiop J Health Dev 26(1): 36–42.

[25] Dzowela T KO, Lgbigbia A (2005) Prevalence of group B Streptococcus colonization in antenatal women at the Queen Elizabeth Central Hospital Blantyre-a preliminary study. Malawi Med J 17: 97–99.

[26] Moyo SR, Mudzori J, Tswana SA, Maeland JA (2000) Prevalence, capsular type distribution, anthropometric and obstetric factors of group B Streptococcus (Streptococcus agalactiae) colonization in pregnancy. The Central African journal of medicine 46: 115–120. 11210331 10.4314/cajm.v46i5.8533

[27] Gilbert GL, Hewitt MC, Turner CM, Leeder SR (2002) Epidemiology and predictive values of risk factors for neonatal group B streptococcal sepsis. Aust N Z J Obstet Gynaecol 42: 497–503. 12495094 10.1111/j.0004-8666.2002.00497.x

[28] Zamzami TY, Marzouki AM, Nasrat HA (2011) Prevalence rate of group B streptococcal colonization among women in labor at King Abdul-Aziz University Hospital. Archives of gynecology and obstetrics 284: 677–679. 21079981 10.1007/s00404-010-1752-2

[29] Baker CJ, Barrett FF, Yow MD (1975) The influence of advancing gestation on group B streptococcal colonization in pregnant women. American journal of obstetrics and gynecology 122: 820–823. 1096618 10.1016/0002-9378(75)90721-8

[30] Slotved HC, Kong F, Lambertsen L, Sauer S, Gilbert GL (2007) Serotype IX, a Proposed New Streptococcus agalactiae Serotype. Journal of clinical microbiology 45: 2929–2936. 17634306 10.1128/JCM.00117-07 PMC2045254

[31] Margarit I, Rinaudo CD, Galeotti CL, Maione D, Ghezzo C, et al (2009) Preventing bacterial infections with pilus-based vaccines: the group B streptococcus paradigm. The Journal of infectious diseases 199: 108–115. 19086816 10.1086/595564

[32] Edwards MS, Gonik B (2013) Preventing the broad spectrum of perinatal morbidity and mortality through group B streptococcal vaccination. Vaccine 31 Suppl 4: D66–71. 23200934 10.1016/j.vaccine.2012.11.046

