# Supplementary material for: Serotype-Specific Acquisition and Loss of Group B Streptococcus Recto-Vaginal Colonization in Late Pregnancy
Source: PLoS One. 2014 Jun 30;9(6):e98778. doi: 10.1371/journal.pone.0098778 (PMC4076185; doi:10.1371/journal.pone.0098778)
Supplement: File S1 — (DOCX) [file pone.0098778.s001.docx]

Table S1. Distribution of serotype associated with GBS recto-vaginal colonization.

| Serotype | Site | Number of isolates per visit ^a^ | | | |
| --- | --- | --- | --- | --- | --- |
|  |  | Visit-1 | Visit-2 | Visit-3 | Visit-4 |
|  |  | n=227 | n=211 | n=175 | n=152 |
| Ia | Vaginal only | 36 (15.9%) | 31 (14.7%) | 29 (16.6%) | 19 (12.5%) |
|  | Rectal only | 29 (12.8%) | 26 (12.3%) | 18 (10.3%) | 18 (11.8%) |
|  | Rectal and vaginal | 29 (12.8%) | 23 (10.9%) | 24 (13.7%) | 18 (11.8%) |
|  | Overall | 94 (41.4%) | 80 (37.9%) | 71 (40.6%) | 55 (36.2%) |
| Ib | Vaginal only | 1 (0.4%) | 0 (0.0%) | 2 (1.1%) | 1 (0.7%) |
|  | Rectal only | 4 (1.8%) | 4 (1.9%) | 0 (0.0%) | 3 (2.0%) |
|  | Rectal and vaginal | 3 (1.3%) | 2 (0.9%) | 2 (1.1%) | 3 (2.0%) |
|  | Overall | 8 (3.5%) | 6 (2.8%) | 4 (2.3%) | 7 (4.6%) |
| II | Vaginal only | 6 (2.6%) | 5 (2.4%) | 3 (1.7%) | 1 (0.7%) |
|  | Rectal only | 4 (1.8%) | 4 (1.9%) | 6 (3.4%) | 6 (3.9%) |
|  | Rectal and vaginal | 7 (3.1%) | 4 (1.9%) | 4 (2.3%) | 4 (2.6%) |
|  | Overall | 17 (7.5%) | 13 (6.2%) | 13 (7.4%) | 11 (7.2%) |
| III | Vaginal only | 17 (7.5%) | 19 (9.0%) | 12 (6.9%) | 11 (7.2%) |
|  | Rectal only | 21 (9.3%) | 24 (11.4%) | 24 (13.7%) | 17 (11.2%) |
|  | Rectal and vaginal | 33 (14.5%) | 23 (10.9%) | 25 (14.3%) | 25 (16.4%) |
|  | Overall | 71 (31.3%) | 66 (31.3%) | 61 (34.9%) | 53 (34.9%) |
| IV | Vaginal only | 3 (1.3%) | 2 (0.9%) | 1 (0.6%) | 0 (0.0%) |
|  | Rectal only | 3 (1.3%) | 2 (0.9%) | 3 (1.7%) | 2 (1.3%) |
|  | Rectal and vaginal | 3 (1.3%) | 3 (1.4%) | 1 (0.6%) | 1 (0.7%) |
|  | Overall | 9 (4.0%) | 7 (3.3%) | 5 (2.9%) | 3 (2.0%) |
| V | Vaginal only | 7 (3.1%) | 11 (5.2%) | 3 (1.7%) | 4 (2.6%) |
|  | Rectal only | 10 (4.4%) | 9 (4.3%) | 7 (4.0%) | 5 (3.3%) |
|  | Rectal and vaginal | 9 (4.0%) | 13 (6.2%) | 8 (4.6%) | 9 (5.9%) |
|  | Overall | 26 (11.5%) | 33 (15.6%) | 18 (10.3%) | 18 (11.8%) |
| IX | Vaginal only | 1 (0.4%) | 4 (1.9%) | 0 (0.0%) | 0 (0.0%) |
|  | Rectal only | 1 (0.4%) | 1 (0.5%) | 1 (0.6%) | 0 (0.0%) |
|  | Rectal and vaginal | 0 (0.0%) | 1 (0.5%) | 2 (1.1%) | 5 (3.3%) |
|  | Overall | 2 (0.9%) | 6 (2.8%) | 3 (1.7%) | 5 (3.3%) |

^a^Percent serotype distribution was calculated using the total number of isolates at each visit as the denominator.

Table S2. Patterns of GBS recto-vaginal colonization overall, and by individual serotypes (n=507).

| **Code*** | Any Serotype | Serotypes | | | | | | |
| --- | --- | --- | --- | --- | --- | --- | --- | --- |
|  |  | Ia | Ib | II | III | IV | V | IX |
| **-,-,-,-** | 255(50.3%) | 387(76.3%) | 493(97.2%) | 485(95.7%) | 414(81.7%) | 500(98.6%) | 471(92.9%) | 500(98.6%) |
| **-,+,-,-** | 14(2.8%) | 10(2.0%) | 0(0.0%) | 2(0.4%0 | 7(1.4%) | 0(0.0%) | 5(1.0%) | 3(0.6%) |
| **-,-,+,-** | 13(2.6%) | 11(2.2%) | 1(0.2%) | 1(0.2%) | 9(1.8%) | 0(0.0%) | 2(0.4%) | 0(0.0%) |
| **-,-,-,+** | 27(5.3%) | 12(2.4%) | 3(0.6%) | 2(0.4%) | 10(2%) | 0(0.0%) | 7(1.4%) | 1(0.2%) |
| **-,+,+,-** | 8(1.6%) | 4(0.8%) | 0(0.0%) | 0(0.0%) | 2(0.4%) | 1(0.2%) | 0(0.0%) | 0(0.0%) |
| **-,+,+,+** | 14(2.8%) | 6(1.2%) | 2(0.4%) | 2(0.4%) | 1(0.2%) | 0(0.0%) | 5(1.0%) | 1(0.2%) |
| **-,-,+,+** | 9(1.8%) | 4(0.8%) | 0(0.0%) | 2(0.4%) | 6(1.2%) | 0(0.0%) | 1(0.2%) | 0(0.0%) |
| **-,+,-,+** | 4(0.8%) | 2(0.4%) | 0(0.0%) | 0(0.0%) | 2(0.4%) | 0(0.0%) | 1(0.2%) | 0(0.0%) |
| **+,-,-,-** | 29(5.7%) | 23(4.5%) | 4(0.8%) | 3(0.6%) | 13(2.6%) | 2(0.4%) | 4(0.8%) | 0(0.0%) |
| **+,+,-,-** | 20(3.9%) | 10(2.0%) | 2(0.4%) | 3(0.6%) | 4(0.8%) | 1(0.2%) | 3(0.6%) | 0(0.0%) |
| **+,+,+,-** | 17(3.4%) | 7(1.4%) | 0(0.0%) | 0(0.0%) | 6(1.2%) | 0(0.0%) | 3(0.6%) | 0(0.0%) |
| **+,+,+,+** | 70(13.8%) | 21(4.1%) | 1(0.2%) | 4(0.8%) | 27(5.3%) | 3(0.6%) | 2(0.4%) | 2(0.4%) |
| **+,-,+,-** | 10(2.0%) | 5(1.0%) | 0(0.0%) | 2(0.4%) | 0(0.0%) | 0(0.0%) | 1(0.2%) | 0(0.0%) |
| **+,+,-,+** | 7(1.4%) | 1(0.2%) | 1(0.2%) | 0(0.0%) | 2(0.4%) | 0(0.0%) | 2(0.4%) | 0(0.0%) |
| **+,-,-,+** | 4(0.8%) | 2(0.4%0 | 0(0.0%) | 0(0.0%) | 2(0.4%) | 0(0.0%) | 0(0.0%) | 0(0.0%) |
| **+,-,+,+** | 6(1.2%) | 2(0.4%) | 0(0.0%) | 1(0.2%) | 2(0.4%) | 0(0.0%) | 0(0.0%) | 0(0.0%) |

Code*: visit-1,visit-2,visit-3,visit-4

Groups: Noncarrier: (-,-,-,-),

Transient carrier: (-,+,-,-) (-,-,+,-) (-,-,-,+) (+,-,-,-)

Intermitent carrier: (-,+,+,-) (-,+,+,+) (-,-,+,+) (-,+,-,+) (+,+,-,-) (+,+,+,-) (+,-,+,-) (+,+,-,+) (+,-,-,+) (+,-,+,+)

Persistent carrier: (+,+,+,+)

"-" Negative for GBS," +"Positive for GBS
